# Tardigrade communities in pristine, drained and restored pine mire forests

**DOI:** 10.1186/s12862-025-02458-9

**Published:** 2025-11-21

**Authors:** Hennariikka Mäenpää, Merja Elo, Janne S. Kotiaho, Emma Meriläinen, Sara Calhim

**Affiliations:** 1 Department of Biological and Environmental Science, University of Jyväskylä, P.O. Box 35, Jyväskylä, FI-40014 Finland; 2 Nature solutions unit, Finnish Environment Institute, Survontie 9 A, Jyväskylä, Finland; 3 School of Resource Wisdom, University of Jyväskylä, P.O. Box 35, Jyväskylä, FI-40014 Finland

**Keywords:** Community ecology, Micrometazoans, Mosses, Peatlands, Peatland drainage, Restoration, Water bears

## Abstract

Restoration has become one of the key measures in improving the state of biodiversity. However, restoration outcomes are in many cases not well understood, and species communities have not been monitored long enough to determine whether they can or cannot recover completely to the pristine reference state. So far, it is particularly poorly known how the communities of microscopic fauna vary within and between pristine, drained and restored peatland habitats. We collected 270 moss samples from a replicated restoration experimental set up to study whether tardigrade communities differ between pristine, drained and restored pine mire forests. In addition, we estimated the associations between tardigrade genera and the moss type they live in. We found a weak pattern of tardigrade occupancy probability being lower in drained and restored sites than in pristine sites. Furthermore, some tardigrade genera were less likely to occur at the drained and restored (11 to 16 years after restoration) sites when compared to pristine sites, although no significant differences in community compositions between treatments were found. We found notable within site variation, which is indicative of high patchiness in tardigrade distribution. We also found strong associations between some of the moss types and tardigrade occurrences. Therefore, tardigrade occurrence seems to be more linked to microhabitat changes, such as mosses, than treatment of the sites. Although the differences between treatments were small, our results show that drained and restored pine mire forests may provide less suitable habitat for tardigrades than the pristine sites do. Favorable habitat conditions for tardigrades in these ecosystems are likely to arise from combinations of large- (e.g., hydrology) and small-scale (moss type) environmental variables that are both affected by drainage and restoration.

**Supplementary Information:**

The online version contains supplementary material available at 10.1186/s12862-025-02458-9.

## Background

Ecological restoration of degraded ecosystems is becoming increasingly important in conserving biodiversity and improving ecosystem functions [1]. Among these ecosystems are boreal peatlands that are degraded by drainage for forestry, agriculture and peat extraction [2, 3]. As moss rich and relatively wet environments, peatlands offer habitats for tardigrades – micrometazoans that are most often found from moist terrestrial habitats and play an important role in the soil food web dynamics [4]. Despite the increasing amount of research on tardigrades during the past few decades, their ecology and habitat preferences remain largely unknown [5]. Moss dwelling tardigrades in peatland habitats remain particularly poorly studied, but it seems they are a common part of the peatland soil fauna, and their abundance and community composition vary across different peatland types and microhabitats [6]. Next to nothing is known about how tardigrade communities are affected by peatland drainage and restoration.

Boreal peatlands include a diversity of habitat types that are congruent by their relative wetness and formation of peat [7]. Peatlands serve as habitats for several species some of which are adapted to certain peatland habitat types, especially among invertebrates [8, 9]. Peatland drainage for forestry includes digging ditches that become the main water flow channels and prevent the water from the catchment area from spreading evenly across the peatland surface. This decreases the water table level which induces timber growth, changes the peat chemistry and composition which influence the temperature and humidity conditions of soil and plant communities dramatically [10–12]. Consequently, species communities tend to shift towards more forest like or generalist species, which is well documented for vegetation. For example, feather mosses such as *Pleurozium schreberi* become more abundant [13, 14]. These changes in habitat conditions and species communities may not happen evenly across the peatland area and may be more notable closer to the ditches [15]. At the landscape level, peatland drainage decreases habitat heterogeneity and biodiversity as peatland habitats become more similar [12].

Restoration of forestry-drained peatlands aims at recovering the pre‒disturbance function and structure (e.g., hydrology, nutrient fluxes and species composition) of peatlands by blocking the ditches to raise the water table level and cutting down trees to reduce evapotranspiration and shading. Restoration is generally an efficient way to increase biodiversity. For example, assemblages of invertebrates, such as *Odonata* [16] and ants [17] start to recover relatively fast. However, restoration rarely succeeds in the full recovery of the pristine habitat species composition during monitored timeframes [12, 14, 18]. The recovery of species happens slowly and gradually, and the restoration outcomes remain not well understood and differ between peatland types and sites [14, 18]. Moreover, the recovery may vary within peatland sites depending on, e.g., the proximity of the ditches [11, 15] especially if drainage has caused peat subsidence near the ditches.

Tardigrades are aquatic in a sense that they are active and reproduce only when surrounded by water, but many species are adapted to survive extreme conditions, e.g., desiccation and freezing, by undergoing a reversible dormant state, cryptobiosis [19–21]. Changes in tardigrade communities and life history traits have been related to, e.g., temperature and moisture [22–25], altitude [26, 27], substrate type [22, 28, 29], and available food resources [5]. From the perspective of tardigrades, the alterations in microclimatic conditions arise from, e.g., type of the material they inhabit, microtopography, vegetation and variation in abiotic factors in the environment. For example, in mosses the water retention capacity and thermal properties vary across moss species with different growth and life forms [30, 31]. The exposure to abiotic factors such as sunlight, wind and ambient relative humidity is in turn determined by the environment. Therefore, the overall favorable conditions for tardigrades are likely affected by combinations of large- and small-scale environmental factors [6, 22, 28, 29].

The changes in habitat conditions, especially in water table level and moss species communities, caused by peatland drainage may shift tardigrade communities towards more desiccation tolerant species. Even though the ability to withstand extreme conditions together with passive dispersal strategies (e.g., by wind and runoff water) have enabled tardigrades to colonize variety of different habitat types, it is also known that tardigrade communities may be altered by several different types of disturbances in their environment. For example, tardigrade community composition and population densities have shown to vary from their natural state reference habitats because of forest clear-cutting [29], pollution [32–34], and urbanization and agriculture [35].

In this paper, we study the variation in tardigrade genera in pristine, drained and restored pine mire forests. Specifically, we ask (1) whether the probability of tardigrade occupancy and abundance differ across treatments (i.e., pristine, drained, restored), and (2) if these differences in tardigrade occupancy and abundance are more notable closer to the ditches in drained and restored sites, and (3) whether the occupancy and abundance of tardigrade genera are more associated with certain moss types. First, we expect to find that tardigrades are more likely to occur and are more abundant at the drained sites than at the pristine sites. We expect this because moss communities and habitat conditions of drained sites resemble more drier peatland habitat types that according to our preliminary studies are more tardigrade rich [6]. In addition, we expect that the post‒restoration increase in water table level and recovery of moss species results tardigrade communities at the restored sites to resemble more those at the pristine sites than those at the drained sites [10, 13, 14]. Second, we expect that tardigrade communities at the drained and restored sites are most divergent from those at the pristine sites closest to the ditch, as is the case for vegetation [15]. Finally, we expect to find higher probability for tardigrade occupancy and abundance in Hypnales mosses than in the subgenera of *Sphagnum* mosses based on earlier studies on moss living tardigrades [6, 22, 29, 36, 37].

## Methods

### Study sites

We selected 15 peatland sites in Finland, where 70% of the land area is covered by forests and over half of the original peatland area has been drained for forestry [12, 38]. The sites are situated in the middle and southern boreal biogeographical zones (Supplementary file 1) and are part of the Finnish peatland restoration monitoring network and are governed by the Finnish Metsähallitus [14]. The sites represent three treatments: (1) five relatively pristine sites with no drainage, (2) five sites that have been drained for forestry during 1960 s and 1970 s and were not restored, and (3) five sites that have been drained for forestry during 1960 s and 1970 s and restored between years 2007‒2012 by filling in and damming the ditches and cutting the trees grown after drainage.

All sites were tall-sedge pine mire forests. We chose to sample tall-sedge pine mire forests for two main reasons: (i) our earlier investigations have shown that tardigrades are common in this peatland type and their abundance is relatively even across sites [6]; (ii) vegetation seems to recover relatively well after restoration in these peatland types [12, 14]. Tall-sedge pine mire forests are mesotrophic forested peatlands that represent the medium level of nutrient availability in the continuum of boreal peatland ecosystems [7]. The dominant tree species is pine (*Pinus sylvestris*), sometimes together with sparsely growing Norway spruce (*Picea abies*) and birch (*Betula pubescens*). *Sphagnum* mosses dominate the lower and more moist levels of the surface microtopography (such as *S. fallax*, *S*. *angustifolium*, *S. divinum*, and *S*. *russowii*) and patches of forest mosses (e.g., *Pleurozium schreberi*) and *Dicranum* mosses grow especially on hummocks and tree bases. The understory vegetation consists of tall sedges (e.g., *Carex lasiocarpa*, *Eriophorum vaginatum*). The peat layer is often deep (1–1.5 m), the water table level is below the surface and the dominating microtopographic level is hummock [39].

### Sampling

**Fig. 1:**
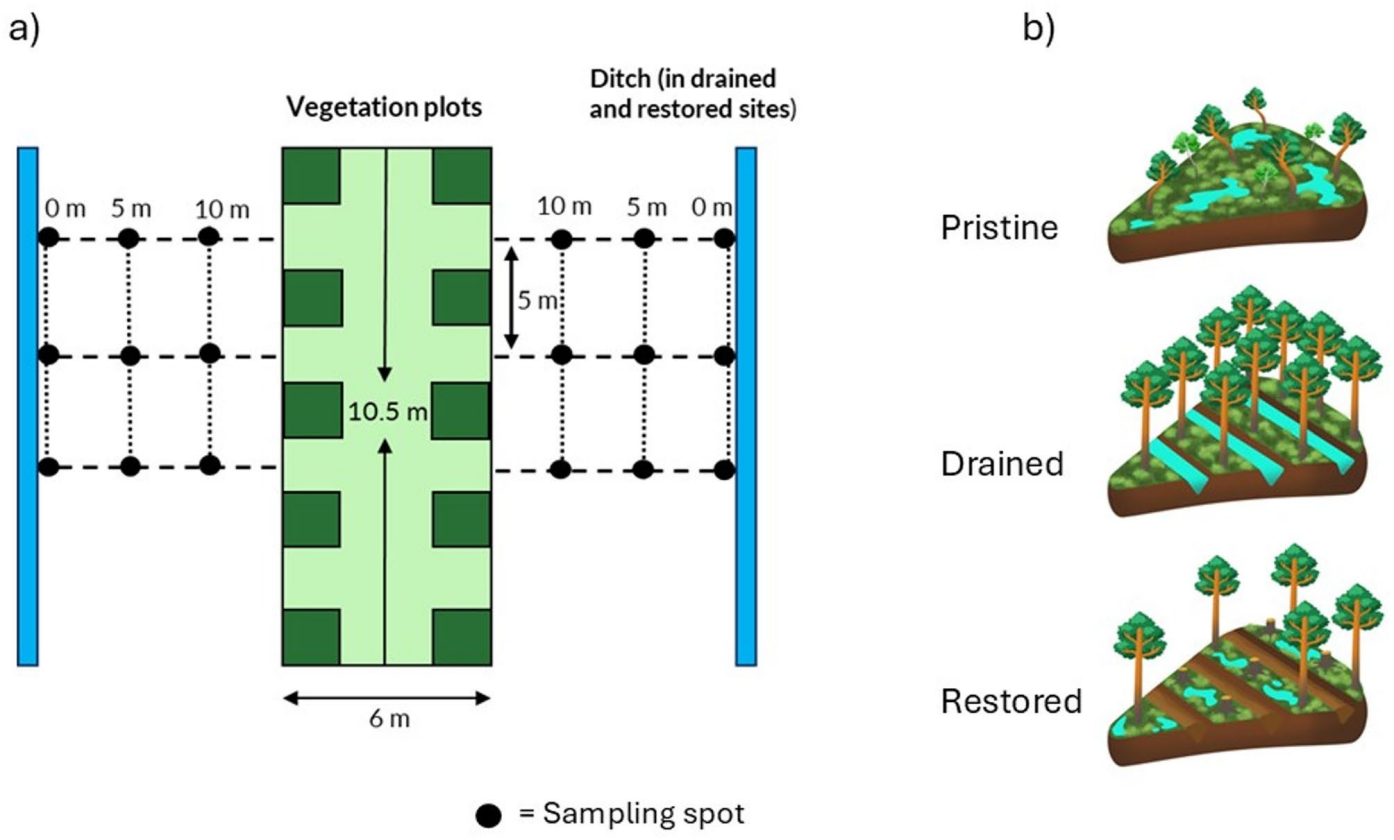
**a** The moss samples were collected along six 15 m sampling lines that were measured perpendicular to permanent vegetation plots at the sites (the plots were not used in this study) at 0 m, 5 m and 10 m distances from the ditches (in drained and restored sites). Corresponding distances were sampled at the pristine sites. **b** The peatland sites were tall-sedge pine mire forests that included five “pristine” sites with no drainage, five “drained” sites that were drained for forestry during the 1960 s and 1970 s and five “restored” sites that were previously drained and had been restored between 2007–2012 by blocking the ditches and cutting down some of the trees

We collected moss samples from the sites in September 2023 (sampling license obtained from the Finnish Metsähallitus Parks and Wildlife services, license number MH2681/2021/1). Each site has ten permanent vegetation monitoring plots (the plots were not used in the study). The plots are situated approximately in the center of ~ 30 m wide area between ditches at the drained and restored sites. We placed three sampling lines five meters apart on both sides of the vegetation plots. We set sampling lines perpendicularly to the vegetation plots so that the end of the sampling line was right next to the ditch in drained and restored sites (~ 15 m from the vegetation plots). We collected moss samples at 0 m, 5 m, and 10 m distances from the ditch at the drained and restored sites. Although there were no ditches at the pristine sites, we sampled corresponding distances (resulting in 6 × 3 = 18 samples for each site, i.e., 270 total) (Fig. 1).

We used metal cylinders (depth = 6 cm, diameter = 9 cm) to collect the moss samples. This ensured that all samples had the same volume of moss. Yet some samples consisted of a single moss species whereas others represented a mixture of different moss species. We stored the moss samples in 1 L sealable plastic bags and placed them into coolers to prevent the temperature from increasing inside the bags to tardigrade lethal readings [40] during transportation.

### Sample processing

We extracted tardigrades from the samples at the University of Jyväskylä with the Baermann wet funnel method with sieves of 1 mm and 24 h extraction duration. We took pieces of moss evenly throughout the sample to cover the 10 cm diameter sieves without stacking the material (one sample/sieve) and stored the remaining samples. When a sample consisted of more than one moss species the part that ended on the sieve represented all the species that were found in the sample. The sieves were then placed into the funnel cones and covered with tap water. After the extraction, we removed the vials attached to the end of the funnels and replaced the water with 70% ethanol to store the extracted animals. The moss was collected from the sieves and dried at 60 °C for 48 h and weighed at precision of 1 mg (Mettler Toledo XS204 DeltaRange).

We identified the moss species from the entire collected samples in a laboratory with a stereomicroscope (Olympus SZX9, 10× magnification) (identification by H.M.). We grouped the moss species into seven groups to best describe their ecology and substrate characteristics based on their growth and life forms [7, 30, 41]. Mosses with *Sphagnum* growth form were grouped into three subgenera: (1) *Sphagnum* are big and robust and grow on hummocks: *S. divinum* and *S. palustre*, (2) *Cuspidata* are small to medium sized that grow on lower and more wet layers: *S. angustifolium*, *S. fallax*, *S. balticum*, *S. riparium* and *S. flexuosum*, and (3) *Acutifolia* are small to medium sized an grow on hummocks: *S. capillifolium*, *S. fuscum*, *S. girgensohnii*, *S. russowii* and *S. squarrosum*. Mosses with acrocarpous growth form were grouped based on genus: (4) *Dicranum: D. polysetum*, *D. scoparium*, *D. undulatum*, (5) *Aulacomnium: A. palustre*, and (6) *Polytrichum: P. commune*,* P. strictum*. Mosses with pleurocarpous growth form were grouped by order into Hypnales: *Pleurozium schreberi*, *Calliergon cordifolium* and *Warnstrofia fluitans*.

We counted tardigrades from the samples under the stereomicroscope. Because of the high number of samples and individuals, we mounted a maximum of randomly selected 50 tardigrades (if present) from each sample in Hoyer’s medium for identification. This number was chosen based on the number of specimens that could be analyzed with the available time and resources, which is common practice in tardigrade studies [42, 43]. By choosing this number we aimed at approximately 50% identification success out of all collected tardigrades. We identified the mounted specimens to genus level under a phase contrast microscope (Zeiss AXIO, 100× magnification) (identification by H.M. and E.M.). We did not find any tardigrade eggs, which is probably due to our extraction method. Therefore, we chose the genus-level identification, which was the most reliable since species-level identification for many of the found genera would require detailed analysis of eggs.

Specimens of each collected moss species and identified tardigrade specimens will be deposited to the Natural history museum of the University of Jyväskylä.

### Statistical methods

We analyzed the differences in tardigrade communities across treatments (pristine, drainage and restoration) and sampling distances from the ditches (0 m, 5 m and 10 m) with Bayesian joint species distribution modelling: the Hierarchical Modelling of Species Communities framework (HMSC) [44, 45]. We ran two models with the same study design. First, we modelled the occupancy (presence/absence) of tardigrades and each genus that had at least ten occupancies in the samples (12 genera). Second, we modelled the overall abundance of tardigrades and the relative abundance of the genera conditional to occupancy. The relative abundance of each genus was calculated in relation to the total number of tardigrades in a sample.

Because our sampling scheme was hierarchical, we included ‘site’ as a spatially explicit random effect by using site coordinates, and ‘line’ (nested within a site), and ‘sample ID’ (nested within a line) as unstructured random effects. As fixed effects, we included moss dry weight (g) (a continuous variable) to control for the differences in the sample amount in the funnels, treatment (a three-level factor: pristine, drained and restored), distance (a three-level factor: 0 m, 5 m, and 10 m) and the interaction of treatment and distance.

The occupancy of tardigrades and each genus was included in the first model with probit distribution. The model also included the occupancy of each moss type (presence/absence) in a sample with probit distribution. The number of different moss types within a sample was included with poisson distribution. The second model included the overall tardigrade abundance and relative abundance of each genus conditionally to occupancy (log-transformed, normalized to zero mean and unit variance within each genus) with a normal distribution. Moss types and number of moss types in a sample were included similarly as in the first model.

We ran the models using the Bayesian framework with Gibbs Markov chain Monte Carlo (MCMC) sampling. We fitted the model using four independent MCMC chains of 300 000 iterations. For each chain, the first 50 000 iterations were discarded as a burn-in. The remaining 250 000 iterations were thinned by 1000, yielding in 250 posterior samples per chain and 1000 samples in total across the chains. We assessed the effective size of the posterior sample and potential scale reduction values to estimate the MCMC chain convergence (Supplementary file 2). We assessed model fitting by estimating the difference between explanatory power and predictive power by two-fold cross validation with Tjur’s R^2^ (genera and tardigrade overall occupancy and occupancy of moss groups), R^2^ (genera and total tardigrade abundance) and SR^2^ (number of moss groups) (Supplementary file 3).

Based on the fitted models, we predicted the probability of tardigrade occupancy and total abundance and probability of occupancy and abundance of each genus across different sampling distances in different treatments. We used these predictions to calculate measures to interpret differences in tardigrade occupancy and abundance in relation to treatment and distance. To standardize the amount of substrate, the predictions were calculated for the minimum weight of moss in the samples (0.305 g).

First, we calculated the difference in the probability of occupancy (O) of tardigrades and each genus between drained and pristine sites:

1 $$\text{Diff}_{\text{Drained} - \text{Pristine}} = \text{O}^{\text{D}} - \text{O}^{\text{P}}$$

Where ‘O^’^ refers to tardigrade occupancy, and in the superscript ‘D’ to drained and ‘P’ to pristine. We calculated the difference similarly between restored and pristine and restored and drained sites.

Second, we calculated the difference in occupancy within sampling distances between treatments:

2 $${\text{Diff}}_{\text{Drained}^{10} -\text{Pristine}^{10}}=\text{O}^{\mathrm D10}-\text{O}^\text{P10}$$

3 $${\text{Diff}}_{\text{Drained}^{5} -\text{Pristine}^{5}}=\text{O}^{\mathrm D5}-\text{O}^\text{P5}$$

4 $${\text{Diff}}_{\text{Drained}^{0} -\text{Pristine}^{0}}=\text{O}^{\mathrm D0}-\text{O}^\text{P0}$$

and repeated these calculations between other treatments by subtracting pristine from restored and drained from restored sites.

Third, we calculated the difference between five- and ten-meter distances within treatments:

5 $${\text{Diff}}_{\text{Pristine}^{5} -\text{Pristine}^{10}}=\text{O}^{\mathrm P5}-\text{O}^\text{P10}$$

and zero- and ten-meter distances:

6 $${\text{Diff}}_{\text{Pristine}^{0} -\text{Pristine}^{10}}=\text{O}^{\mathrm P0}-\text{O}^\text{P10}$$

and did similar calculations for drained and restored sites.

We repeated these calculations [1]- [6] for the tardigrade overall abundance and the relative abundance of each genus.

We calculated the median and posterior probability for the median being larger/smaller than zero for all the measures from Eqs. (1)-(6). If the posterior probability (pp) was > 95% we considered the measure to have high support for the median being positive/negative.

For visualizing variation in community composition across treatments and distances from the ditch, we used non-metric multidimensional scaling (NMDS; function metaMDS). The analysis was based on tardigrade occupancy data using the incidence-based Jaccard dissimilarity index.

We estimated the associations between moss groups and tardigrade genera by visualizing residual between taxa associations with probability of 0.95.

We used R version 4.4.1 [46] and the R-packages Hmsc [47], ggplot2 [48], dplyr [49], vegan [50], and patchwork [51].

## Results

We found a total of 5943 tardigrades from 86% of the samples (231/269 processed) that had tardigrades with numbers ranging between 1 and 218 individuals. We identified successfully 3081 specimens (52% of the collected tardigrades) into 18 genera. Of these genera, 12 occurred in more than 10 samples and were included in the models. A list of the identified genera and moss types within samples can be found in Supplementary file 4.

**Fig. 2:**
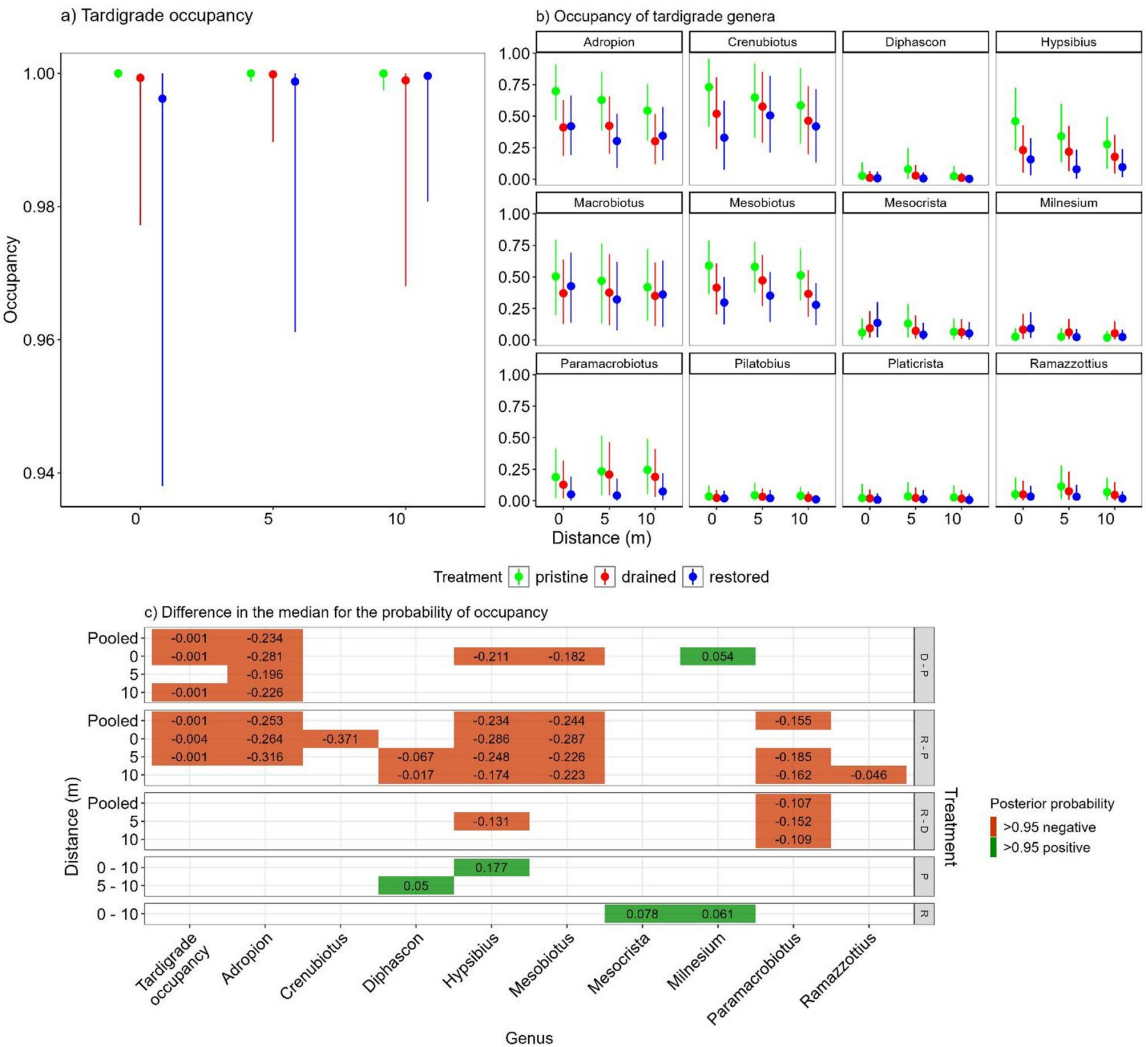
The model estimates for the median posterior probability with the minimum and maximum value of high density continuous interval (HDCI) for tardigrade overall occupancy (**a**) and occupancy of individual genera (**b**) across the treatments and three sampling distances from the ditch. The lower panel (**c**) shows the measures that were estimated to have at least 0.95 posterior probability of positive (green) or negative (red) differences in the posterior median between treatments. The righthand panel refers to treatments (P = pristine, D = drained, R = Restored) and their order within panel to the direction of subtraction. The lefthand panel refers to sampling distances from the ditch. All calculated posterior median differences and their posterior probabilities can be found in Supplementary file 4

The median probability for tardigrade occupancy was high in all treatments (> 0.98) but there was more variation within drained and restored sites (Fig. 2a). The median occupancy was lower at the drained and restored sites when compared to pristine but with a very small difference in the median (0.001), whereas there was no distinct difference between drained and restored sites (Fig. 2c). When considering individual genera, *Adropion* was less likely to occur at all distances and *Hypsibius* and *Mesobiotus* were less likely to occur at the 0 m distance from the ditch at the drained sites than at the pristine sites (Fig. 2b, c). When restored and pristine sites were compared *Adropion* and *Mesobiotus* were less likely to occur at all distances and *Crenubiotus*, *Diphascon*, *Hypsibius*, and *Paramacrobiotus* had a lower probability of occupancy at least at some distances (Fig. 2b, c). The only exception across the genera was *Milnesium* that was more likely to occur at the drained sites than at the pristine sites, especially at the 0 m sampling distance (next to the ditch) with a small difference in the median (0.054) (Fig. 2c). We found very little within treatment variation between the distances in occupancy of individual genera (Fig. 2c). At the pristine sites, *Hypsibius* was more likely to occur at 0 m and *Diphascon* at 5 m than at 10 m sampling distances. In addition, *Mesocrista* and *Milnesium* were more likely to be found at 0 m than 10 m distance from the ditch in the restored sites.

**Fig. 3:**
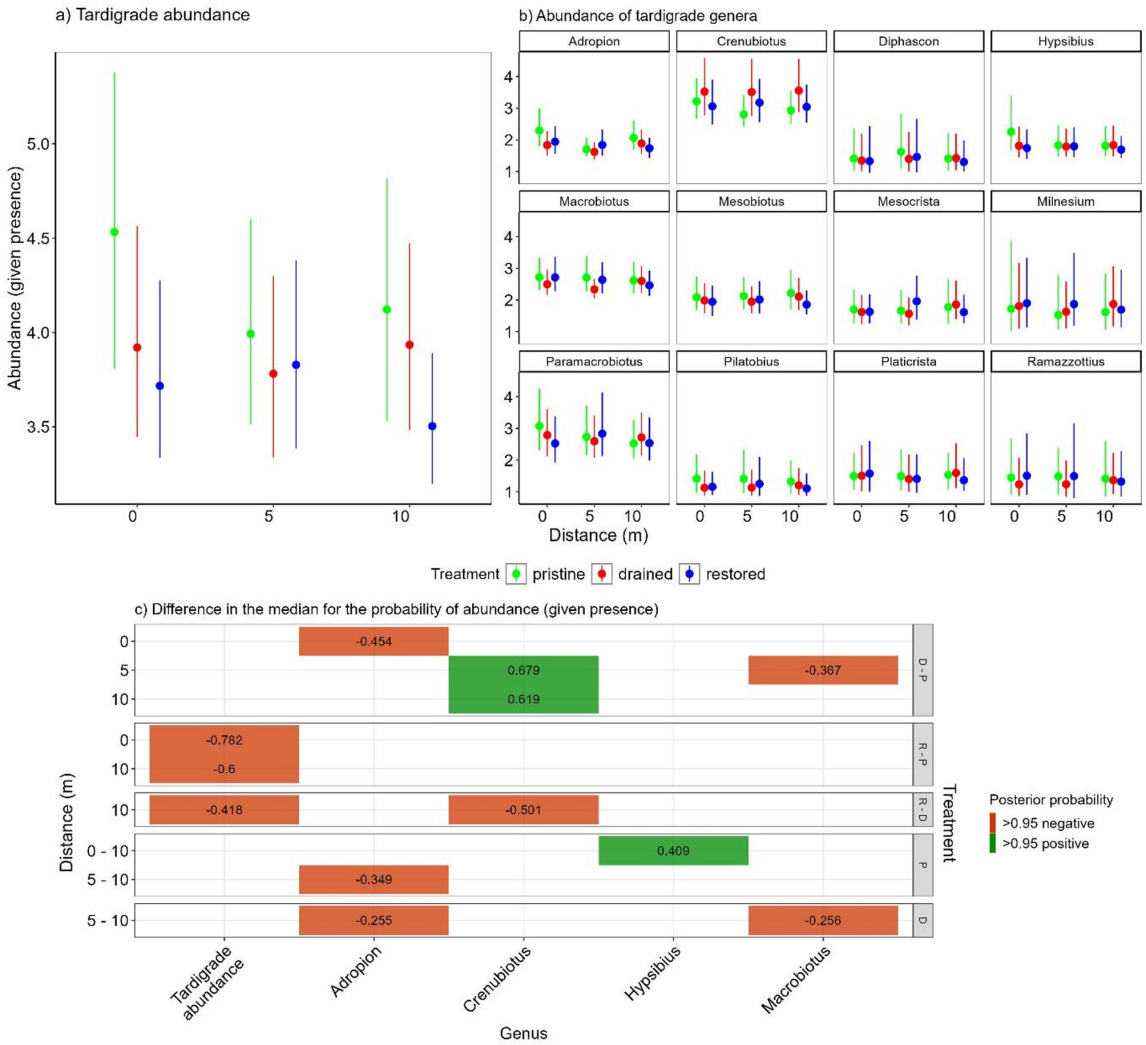
The model estimates for the median posterior probability with the minimum and maximum value of high density continuous interval (HDCI) of tardigrade overall abundance given presence (**a**), and abundance of individual genera when present (**b**) across the treatments and three sampling distances from the ditch. The lower panel shows the measures that were estimated to have at least 0.95 posterior probability of positive (green) or negative (red) differences in the posterior median between treatments (**c**). The abbreviations in the panels are similar as in Fig. 2. All calculated posterior median differences and their posterior probabilities can be found in Supplementary file 4

Given the presence, the overall tardigrade abundance at the drained and restored sites was similar to the pristine sites when all sampling distances were considered (Fig. 3a, c). The abundance was lower at the restored sites at 10 m and 0 m distances than at the pristine and at the 10 m distance when compared to drained (Fig. 3c). Across the genera *Adropion* had lower abundance at 0 m distance and *Macrobiotus* at 5 m distance at the restored sites when compared to pristine sites (Fig. 3b, c). At the drained sites only *Crenubiotus* was found in higher numbers at 10 m and 5 m distances than at the pristine sites.

**Fig. 4:**
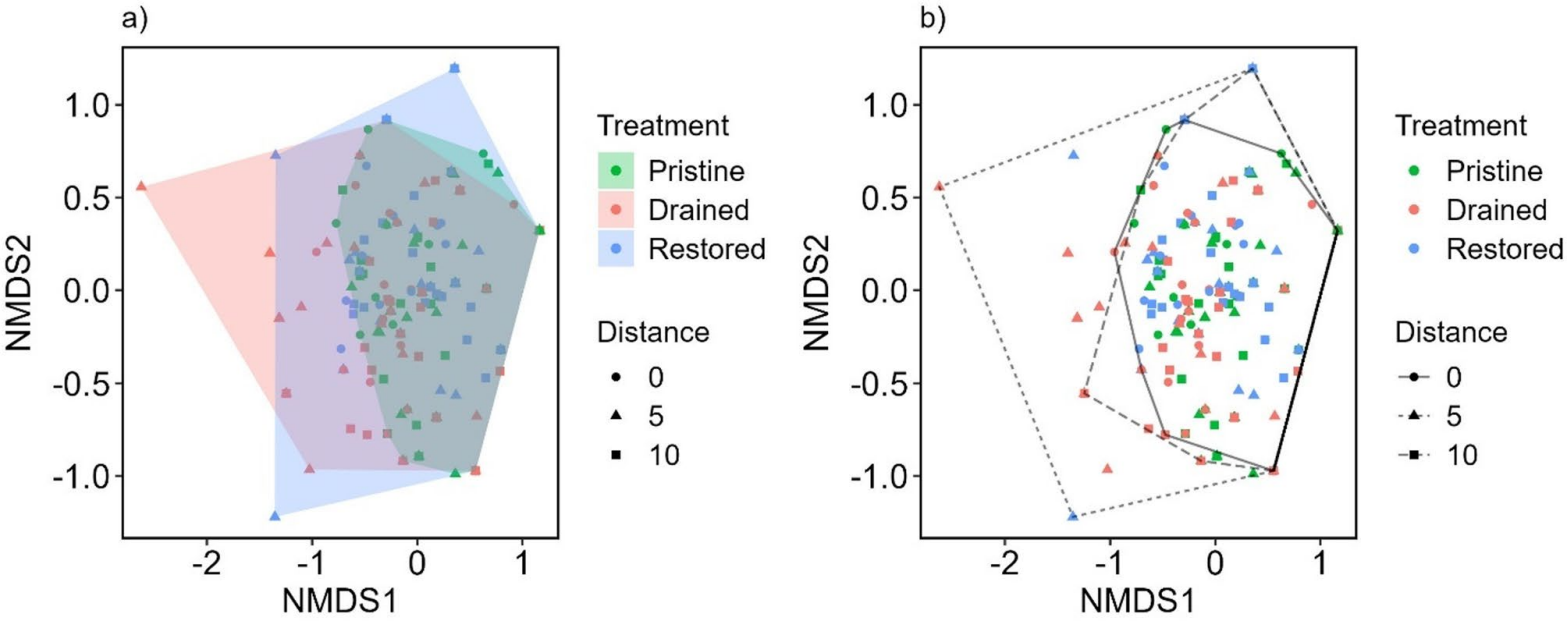
Non-metric multidimensional scaling of community composition across treatments and distances from the ditch. Colored hulls (**a**) represent the three treatments (Pristine, Drained, Restored) and line types (**b**) represent different distances from the ditch (0 m, 5 m, 10 m). Both figures (**a**) and (**b**) were created using the incidence-based Jaccard index

The presence-absence based Jaccard index did not show any distinct differences in tardigrade community composition in relation to treatments or distances from the ditch (Fig. 4).

**Fig. 5:**
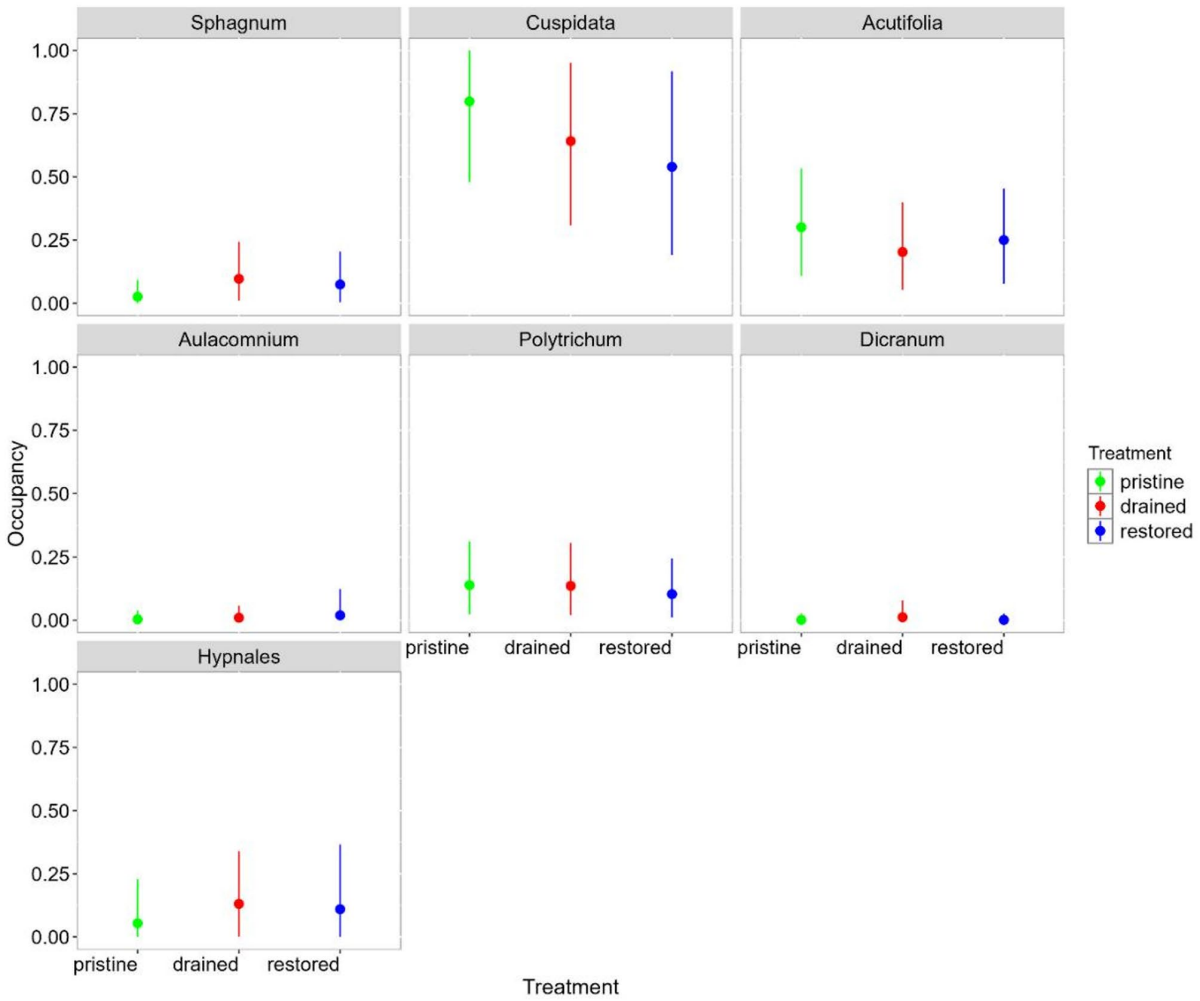
The model estimates for the median occupancy probability of moss types across the treatments

**Fig. 6:**
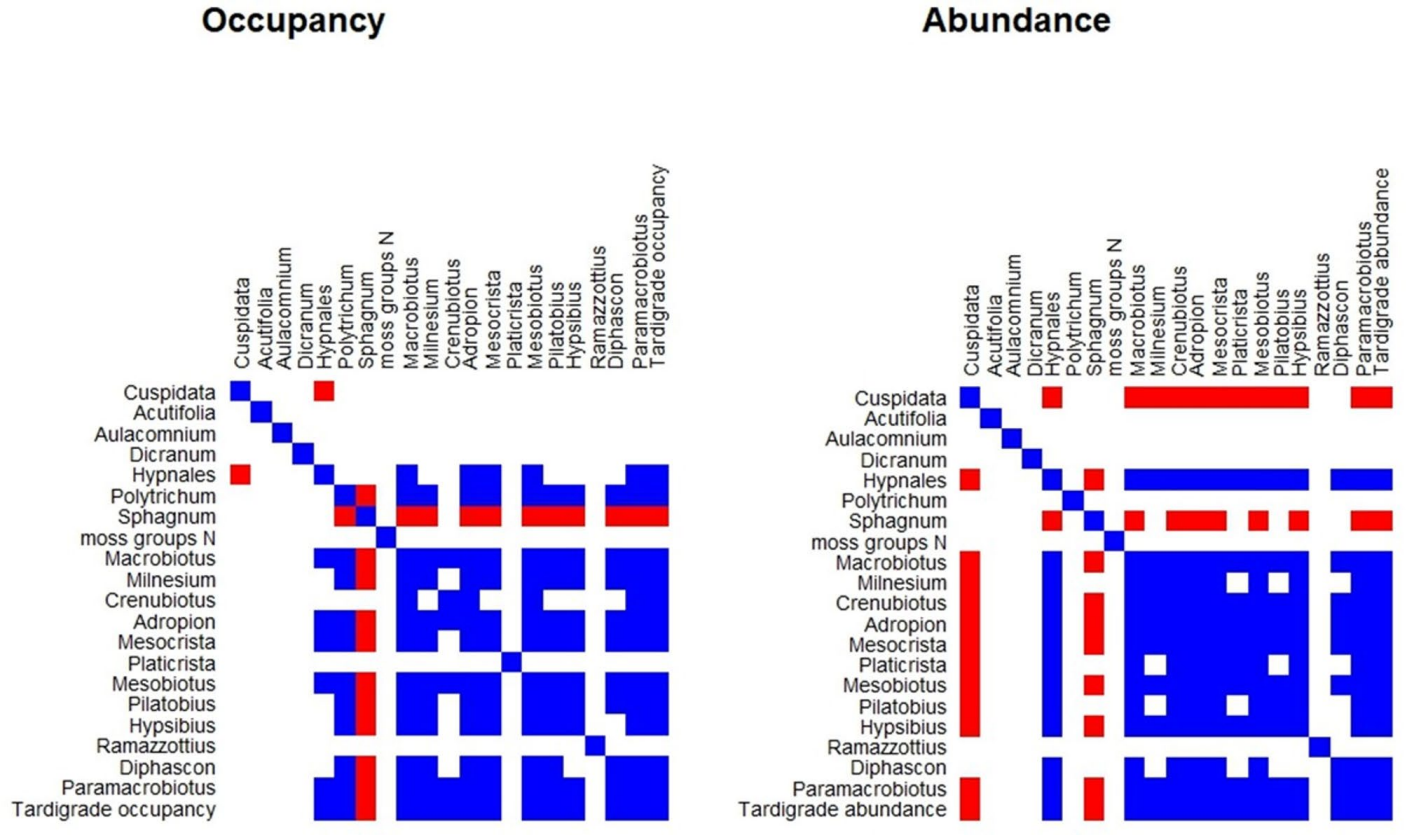
Residual associations between taxa. The first panel corresponds to the model that analyzed tardigrade occupancy and the second panel to the model that analyzed tardigrade abundance conditional to presence. Both model residual associations are based on occupancy of moss groups. In both panels, associations that were estimated positive with at least 0.95 probability are shown in blue, and associations that were estimated negative with at least 0.95 probability are shown in red

The moss group *Cuspidata* had the highest probability of occupancy across all treatments (> 0.5), and it was collected most often from the pristine sites (Fig. 5). Most of the *Sphagnum* and Hypnales mosses were collected from the drained and restored sites. *Polytrichum* mosses were common in all treatment types. The occupancy of tardigrades was positively associated with Hypnales mosses (Fig. 6). Across individual tardigrade genera, occupancy of five were positively associated with Hypnales mosses and nine with *Polytrichum*. The overall abundance of tardigrades and 11 tardigrade genera were positively associated with Hypnales mosses. The only negative association with occupancy of tardigrades was found with *Sphagnum* mosses, whereas abundance was negatively associated with *Cuspidata* and *Sphagnum* mosses. The occupancy and abundance among tardigrade genera were mostly positively associated and none of the genera were negatively associated with each other (Fig. 6).

The variance partition of the models revealed that the random effects explained considerable proportion of the variance in tardigrade occupancy and abundance (Supplementary file 5). Over 80% of the variation in tardigrade total occupancy and abundance was explained by the random effect ‘sample ID’, indicating high irregularity in tardigrade distribution within sites and possibly more pronounced effects of unmeasured factors compared to the predictors. However, these proportions ranged from 6 to 85% for occupancy and 20‒75% for abundance across individual genera. The spatially explicit random effect ‘site’ explained, on average, 11% of the variance in occupancy and 10% of the variance in the abundance of individual genera. These proportions ranged from 2 to 19% for occupancy and 4‒12% for abundance across the genera. Moss weight explained only a small proportion of variation in tardigrade occupancy (4%) and abundance (0.3%).

## Discussion

We studied moss living tardigrade communities in pristine, drained and restored pine mire forests in Finland, and the associations between moss types and tardigrade genera in these habitats. We found small between-treatment differences that showed high uncertainty (Figs. 2 and 3), which nevertheless suggest that drained peatlands offer less suitable habitat for tardigrades than pristine sites. Furthermore, tardigrade occurrence in peatlands that were restored 11–16 years ago does not seem to be closer to pristine references. Although the occupancy probability of some individual tardigrade genera in drained and restored sites differed from the pristine references, the occupancy-based community composition between treatments did not show any notable differences (Fig. 4). If the conditions are favorable for tardigrades to occur, their abundance does not vary notably between treatments or in relation to the proximity of the ditches. The reason why we found such small between-treatment differences was that most of the variation was explained by the random effects in our models (particularly ‘sample ID’), which partly reflects the patchiness of tardigrades in these habitats. This high within site variation may also reflect small sample size relative to the characteristic patchiness of tardigrade occurrence. However, this patchiness is also likely to be related to the strong associations between tardigrades and certain moss types (Fig. 6). Thus, although the management history of the sites matters to some extent, tardigrade occupancy and abundance are also strongly driven by microhabitat variation within the sites, particularly related to the variation in moss types.

### Peatland treatment and habitat conditions

Our results show higher tardigrade occupancy probability in pristine than drained sites with a small difference in the median and high uncertainty. The higher occupancy in pristine sites is contrary to our expectations, since our preliminary investigations have shown that tardigrades are likely to be found in high numbers in relatively dry peatland habitats with high canopy cover, such as drained peatlands [6]. Mosses are more likely to experience occasional desiccation at the drained sites than at the pristine sites, and typically such habitats have high tardigrade population densities and species richness [42, 52, 53]. Conversely, it is also known that repeated or prolonged cryptobiosis may have negative consequences on tardigrade fitness [54–57]. Our results also deviate from some previous research on other micrometazoans, as post-drainage decrease in water level has been found to increase the abundance of, for example, nematodes, mites and collembola [58–60]. Water table level and moisture regimes are among the most important drivers of variation in invertebrate communities of peatlands. For instance, nematode [61–63] and testate amoeba communities [63, 64] are mainly determined by hydrology in different peatland types. To further understand the impact of moisture conditions for tardigrade occurrence in these habitats would require measuring sampling spot specific within moss moisture levels over a longer period.

Similarly, small and uncertain differences were found between restored and pristine sites, suggesting lower tardigrade overall occupancy probability in restored sites. Although community composition did not differ between the treatments according to the NMDS ordination, the occupancy probabilities of some individual genera differed more between pristine and restored sites than between restored and drained sites based on the HMSC model. One possible reason creating varying habitat conditions could be differences in microtopography between restored and pristine sites. Drainage causes significant changes in microtopography by increasing peat compaction and density which flattens the characteristic surface patterns [12, 61, 65]. If the microtopography recovers after restoration it is likely to take a very long time, at least decades [66]. In comparison, pristine pine mire forests have more variation in microtopography, as hummocks rise further above the water table and the lower levels, lawns and hollows, lie between the hummocks [7]. This variation in microtopography creates habitat heterogeneity and provides larger range and area of microhabitats with differences in moisture levels. In addition to moisture, depressions between hummocks provide shelter from the surrounding abiotic conditions, since microtopography plays an important role in the temperature dynamics of peatland surface [67]. For instance, the difference in the temperature in north- and south-facing slopes of hummocks may be up to 13 °C during daytime and south-facing hummocks experience greater diurnal variation [68]. This would mean considerable difference in the within substrate thermal conditions to which many tardigrade species are known to be sensitive [23, 40, 69]. Although human made ditches create microtopographic variation as well, the ditches also create wide openings in the canopy cover and therefore generate very different habitat conditions on the peatland surface compared to natural hollows. However, we did not collect samples from the actual ditches, and it would make an interesting comparison to see how well tardigrades are able to inhabit these artificial land formations.

Since tardigrades utilize passive dispersal strategies (e.g. along wind, runoff water and even other, larger animals), it is unlikely that the between treatment differences would be due to lack of time for tardigrades to disperse to the sites [5, 70]. When conditions are favorable, tardigrades reproduce relatively fast and new generations may be produced within a few weeks [71]. Since the peatland sites were restored 11‒16 years before our sampling, tardigrades would have had plenty of time to migrate and establish abundant populations at the restored sites. The passive distribution has its benefits for microscopic fauna and enables migration even for longer distances, but it is mostly due to chance whether the individuals end up in habitat patches that are suitable for them. Thus, if the area of suitable habitats is low their chances of ending up in them remain low. This is very different from the macroinvertebrates that have been studied in restored peatlands. For example, Odonata and butterflies rely on visual cues and can fly relatively long distances (kilometers) and are able to find suitable waterbodies for laying eggs that emerge relatively soon after restoration [16, 72]. Tardigrades can move approximately 1.98–4.8 mm/min on a smooth, wet and horizontal surface [73]. However, they are unlikely to reach this speed under natural conditions and even small obstacles, such as dry patches, will become dispersal barriers. Thus, they cannot actively migrate between fragmented habitat patches but instead remain dormant and wait for the conditions to improve.

*Milnesium* was the only genus that had higher occupancy probability in drained sites compared to pristine. Furthermore, it was more likely to be found near the old ditches than in the center of the restored sites. Genus *Milnesium* includes some desiccation tolerant species, for example, *M. tardigradum* and *M. inceptum* that are commonly found in mosses that grow in dry habitats [6, 74–76]. *Milnesium* is known to be the only genus that is truly carnivorous that cannot reproduce without animal prey in its diet [77]. They commonly feed on other micrometazoans, such as nematodes, rotifers and smaller tardigrade species. If drainage generally increases the abundance of soil fauna, it is likely that drained peatlands provide more food resources for predatory tardigrades than pristine sites. Altogether, we found very few *Milnesium*, only 55 specimens (out of 3081 identified specimens), and their abundance did not differ between treatments. Thus, it seems that despite the favorable conditions for *Milnesium* to occupy, especially near the ditches in drained and restored sites, they are not able to establish large populations in any of the treatment types. Interestingly, we also found *Thulinius* and *Paramurrayon* in pristine and restored sites, but unfortunately, they occurred only in a few samples and could not be included in the models (Supplementary file 4: Identified genera). *Thulinius* and *Paramurrayon* are widely considered as limnic tardigrade genera because most species described so far have been discovered mainly from freshwater habitats [78–80]. The presence of *Thulinius* and *Paramurrayon* suggests that the elevated water table at restored sites may offer more diverse habitats for tardigrades than drained sites, however, the limited number of observations prevents drawing definitive conclusions.

### Distance to the ditches

We did not detect any gradient in tardigrade overall occupancy or abundance in relation to the proximity of the ditch (or the old ditch) within drained and restored sites. Nevertheless, some of the between-treatment differences were strongest at the 0 m distance, especially between the pristine and restored sites. The variation in tardigrade occupancy at the restored sites was highest next to the old ditches. Interestingly, the probability of tardigrade occupancy and abundance increased slightly at the 0 m sampling distance at the pristine sites compared to 10 m distance, although no statistical support for a strong gradient was found within the treatment. This trend seemed to be present in many genera (e.g., *Adropion*, *Crenubiotus*, *Macrobiotus* and *Paramacrobiotus*) (Figs. 2b and 3b) but only *Hypsibius* had a statistically supported higher probability of occupancy and abundance at the 0 m sampling distances in pristine sites when compared to 10 m sampling distances. This is an interesting observation, since pristine sites do not have a ditch that could cause this pattern. The vegetation plots that were used as references to measure the 15 m sampling lines in pristine peatlands were situated roughly in the middle of the sites. However, since the pristine sites were relatively small the 15 m distance from the vegetation plots (i.e., 0 m sampling distance in our study) were already very close to the edges of the sites. In pristine peatlands, small-scale variation in vegetation regimes and abiotic conditions are common and generally, the edges of peatlands represent habitat type complexes, where fen vegetation shifts gradually to forest vegetation but is hydrologically connected [7, 39, 81]. This shift in habitat conditions could explain some of the patterns we have observed in pristine sites. Furthermore, similar patterns in relation to distance in pristine peatlands have been observed in studies on plant communities [15].

When tardigrades were present, their abundance was lowest at 10 m from the old ditches at the restored sites. The abundance was lower when compared to the 10 m distances at both pristine and drained sites. Generally, the vegetation communities of restored peatlands often resemble more pristine sites further away from the old ditches [15] and the post-restoration recovery in hydrology is slower near the old ditches than at the center of the sites [11, 82]. Given this, the 10 m distances from the old ditches at the restored sites are expected to offer habitat conditions most similar to those of the pristine sites. This is why we anticipated that tardigrade abundance would be closest to that of the pristine reference sites at these sampling spots. Whether the lower abundance at the restored sites is because of the above discussed differences in moisture and microtopography remains unknown. It is typical that the habitat conditions and species communities of restored ecosystems end up resembling something between pristine and drained ecosystems within the monitored timeframe [10, 14, 15, 17, 64, 72, 82]. Altogether, our results suggest that these in-between conditions do not seem to be very suitable for tardigrades.

### Associations between tardigrades and moss types

We found positive residual association between the occupancy and abundance of tardigrades and Hypnales group mosses, as we had predicted. Majority of the Hypnales samples included species *P. schreberi* that was most prevalent in the samples collected from the drained and restored sites. Since these mosses are common in forests and on hummocks of forested peatland habitats, they tend to become more prevalent in peatlands after drainage [13, 14]. Note, however, that *W. fluitans* and *C. cordifolium* that were included in the Hypnales group are not forest species but typical in fens and wetter parts of pine mire forests. *W. fluitans* was present in one sample and *C. cordifolium* was present in three samples that were all collected from the pristine sites. Hypnales mosses have a pleurocarpous growth form, and such mosses have been associated with high tardigrade densities also in previous studies [6, 22, 29, 36, 37]. Pleurocarp mosses have branched stems that lie horizontally close to the surface and form loose mats, so-called wefts [30]. These mosses have a complex, small-leaved architecture that possibly provides high micro-scale habitat diversity within the wefts. Furthermore, the growth and life forms of the mosses are related to their water retention capacity, thermal properties and aeration within the cushions [30, 31]. However, it remains unknown which particular feature in these types of mosses impacts the suitability to host tardigrades. Tardigrade occupancy was also positively associated with *Polytrichum* mosses, which is a genus of mosses with acrocarpous growth form that stand vertically and form turfs on hummocks of forested peatlands. *Polytrichum* mosses grow long, flexible, and hardy stems that extend below the surface. Therefore, it is difficult to say whether the found tardigrades were in the actual moss stems or in the surface material that was lifted with the stems.

*Cuspidata* was the most common moss type in our samples and was collected most often from the pristine sites. *Cuspidata* was negatively associated with tardigrade abundance and with Hypnales mosses. *Cuspidata* mosses are common in the wetter parts of pine mire forests, whereas majority of the Hypnales mosses (mostly *P. schreberi*) in our study were collected from drier habitat patches. Due to their different habitats, these mosses also provide very different living conditions for tardigrades. It is possible that the positive associations between tardigrades and moss types reflect the similarity in habitat requirements of the two, rather than the moss types themselves being more suitable habitats. Certain mosses may grow in habitat conditions that are more suited for tardigrades, which is why they are found in high numbers in these mosses.

It should be noted that these residual associations between taxa were found after the treatment and distance were considered in our models and provide further explanation for the high within site variation in tardigrade distribution. Therefore, moss type is likely a key factor contributing to the variation in tardigrade communities that was attributed to ‘sample ID’ in our models. Nevertheless, our results align with earlier conclusions that the suitable habitat conditions for tardigrades are likely to be determined by combinations of large- and small-scale level environmental variables [22, 28, 29]. The material of the collected samples is often categorized on a very generic level in tardigrade ecology studies and divided into, e.g., bryophytes and lichens. Inclusion of more detailed substrate information in ecological studies would provide further valuable information on the interactions of large- and small-scale level environmental variables on tardigrade distribution and has gained more attention in recent studies [22, 83].

### Implications of the results and methodological considerations

The considerable within site variation (i.e., patchiness) of tardigrade occupancy and abundance, makes it difficult to assess differences between treatments. Although patchiness in tardigrade occurrence is a common problem in tardigrade ecological studies (discussed e.g., in 6, 22), it is very difficult to predict the adequate sample size, especially in highly heterogeneous habitats, such as peatlands. Further large-scale studies are crucial for a better understanding of tardigrade ecology and their responses to environmental changes. Nevertheless, despite patchiness hindering our conclusions, our study serves as an important reference for future research.

It should be noted that the responses of species communities to drainage and restoration are highly ecosystem type and site dependent [14], and this is likely to be true also for tardigrade communities. Tall-sedge pine mire forests vary from oligotrophic to mesotrophic and are in the middle of the continuum of nutrient gradient and wetness [7]. Generally, restoration towards pristine references has been successful in these peatland types [14, 84]. Moreover, post-drainage decrease in water table level may not be as notable as in wetter types [85]. Therefore, it would be interesting to see whether more notable differences in tardigrade communities would be found in some other peatland types. This would also provide further valuable information on tardigrade distribution in different ecosystem types under anthropogenic disturbance. The spatially explicit random effect ‘site’ explained some of the variation in occupancy and abundance, but the explained proportion varied across the genera (Supplementary file 5). One reason for this could be that the transportation, and hence, storage duration of the samples collected from the sites that are further away has been longer [36]. However, we accounted for this before sample extraction by monitoring the sample storage duration and balancing it across the treatments. This ensured that the longer storage duration would not cause bias towards any of the treatments, although some site-specific differences may occur. Furthermore, the between site variation could also reflect latitudinal differences in communities because the distance between northernmost and southernmost sites was over 400 km.

Our results should be interpreted with caution since we identified tardigrades only to genus level. This may leave out some interesting species-level patterns, especially regarding more species rich genera, e.g., *Macrobiotus*. We found very little differences across treatments and distances in this genus. The family Macrobiotidae (including genera *Macrobiotus*, *Mesobiotu*s, *Parmacrobiotus* and *Minibiotus*) is also known to include high variation in anhydrobiotic performance even between closely related species [86]. Species-level identification would likely give us more detailed information regarding the more numerous genera in this study, such as *Macrobiotus*, *Adropion* and *Crenubiotus*. Furthermore, it would undoubtedly be interesting to compare the results of genus- and species-level variation across the treatments to see how well these reflect ecological differences. However, for many tardigrade genera species-level identification is not possible without detailed analysis of their eggs which was not feasible because of our extraction method [36]. In addition, species-level identification of tardigrades is very difficult, and if done incorrectly may lead to misleading conclusions in ecological studies. Therefore, a viable option could be a combination of morphological and molecular methods by adding metabarcoding data [87] to increase accuracy regarding species distribution at least for the most species rich and numerous genera.

Compared to our preliminary investigations and sampling in similar habitat types, the total amount of tardigrades we found was relatively low. The average number of tardigrades across all samples was 28 individuals per moss gram. As a reference, in our former investigations in pine mire forests the average number of tardigrades has been between 33 and 66 individuals per moss gram [6] or even 593 individuals per moss gram [36]. In addition, half of the genera we found had a low overall probability of occupancy. One plausible explanation for the low number of tardigrades could be that the month preceding our sampling (August 2023) was exceptionally warm and rainy. For example, in central Finland close to some of our study sites the precipitation in August 2023 was 93 mm, whereas the average in August over the past ten years before our sampling has been 69 mm [88]. Consequently, some of the peatland sites were very wet during our sampling. Heavy rainfalls may cause the water table level of peatlands to rise notably and affect the sites differently depending on treatment. The changes in peat surface compaction and density caused by drainage affect the water absorption and retention capacity of the peat for a long time after restoration [65, 89]. Because of this, the excess water during heavy rainfall creates pools on the surface in the restored sites whereas in pristine sites the water is mostly absorbed into the peat. Even though tardigrades need water to stay active, many limnoterrestrial species do not favor stagnant water bodies or submerged substrates.

Since our experimental design did not include a possibility for a before-after drainage and restoration sampling, common to all space-for-time substitution studies, it is difficult to say for sure whether the small differences we found are caused by the treatments or some other causes. Additionally, it would be necessary to repeat the sampling over several years after restoration to understand the gradual effects on tardigrade communities. Such repeated sampling would provide valuable information on the succession of tardigrade communities and would tackle the effects of possible variation in the above-mentioned climatic conditions.

## Conclusions

Although we found very small between-treatment differences, this study demonstrates that drained peatlands may not provide similar habitat conditions for tardigrades as pristine reference sites. Furthermore, the habitat conditions of peatlands restored 11‒16 years ago may still resemble more of those at the drained sites than at the pristine reference sites. This indicates that studies on microscopic fauna could provide valuable information on the recovery of restored ecosystems that are not seen in studies on, e.g., macroinvertebrates. Micrometazoans such as tardigrades play a vital role in ecosystem function by contributing to, e.g., nutrient cycling and regulation of microbial populations. Since habitat type preferences of tardigrades are poorly known, pinpointing the actual environmental variables creating the between-treatment differences is difficult. Patchiness in tardigrade occurrence, which was also present in this study, hinders further conclusions and highlights the need for more studies on tardigrade ecology with considerably large sampling effort. Nonetheless, our study shows that tardigrade communities in peatlands are affected by habitat conditions, such as hydrology (large-scale) and moss communities (small-scale) which are both affected by drainage and restoration. However, it is likely that the small-scale changes in microhabitat conditions are more important drivers of the variation in tardigrade communities.

## Supplementary Information

Supplementary Material 1. Map of the study sites.

Supplementary Material 2. Model convergence.

Supplementary Material 3. Model two-fold cross validation.

Supplementary Material 4. Posterior median and posterior probability; List of the identified tardigrade genera; List of the moss types in samples.

Supplementary Material 5. Variance partition.

Supplementary Material 6. Data for Hmsc-models. 

Supplementary Material 7. Script for model definition and model fitting. 

## Data Availability

All datasets analyzed during the current study are provided in Supplementary file 6 and scripts for model definition and fitting the models in Supplementary file 7.
