# Supplementary material for: Tardigrade communities in pristine, drained and restored pine mire forests
Source: BMC Ecol Evol. 2025 Nov 21;25:126. doi: 10.1186/s12862-025-02458-9 (PMC12639931; doi:10.1186/s12862-025-02458-9)
Supplement: Supplementary file 1 — Supplementary Material 1. Map of the study sites. [file 12862_2025_2458_MOESM1_ESM.pdf]

Mäenpää et al. 2025. Supplementary file 1: Map and coordinates of the study sites

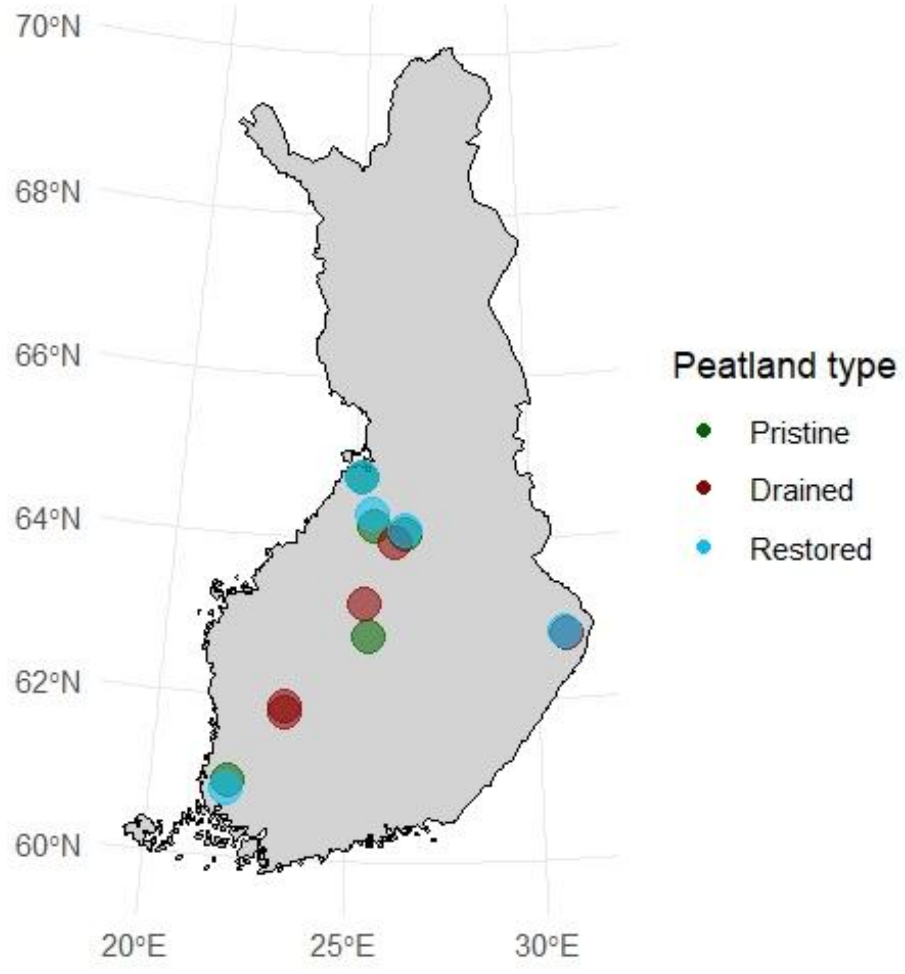

Figure S1. Map of the peatland sites sampled in the study. The sites are situated in Finland in the middle and southern boreal biogeographical zones. The sites represent three treatments: 1) five relatively pristine sites with no drainage (green circles), 2) five sites that have been drained for forestry during 1960s and 1970s and were not restored (red circles), and 3) five sites that have been drained for forestry during 1960s and 1970s and restored between years 2007–2012 by filling in and damming the ditches and cutting the trees grown after drainage (blue circles).

Table S1. A list of the sites with their coordinates and corresponding treatment.

Site ID	Longitudinal (WGS84)	Latitudinal (WGS84)	Treatment
71	60.91734	21.96636	Pristine
72	62.84909	25.45508	Pristine
74	64.72504	25.13348	Pristine
78	64.1166	26.42872	Pristine
79	64.24108	25.5363	Pristine
231	61.88839	23.327	Drained
232	61.90529	23.34581	Drained
233	63.27969	25.36439	Drained
234	63.98686	26.08867	Drained
235	62.83096	30.82252	Drained
61	60.91727	21.92276	Restored
63	62.81371	30.75656	Restored
64	64.7134	25.13613	Restored
68	64.11084	26.44287	Restored
69	64.23908	25.5317	Restored
