# Supplementary material for: Tardigrade communities in pristine, drained and restored pine mire forests
Source: BMC Ecol Evol. 2025 Nov 21;25:126. doi: 10.1186/s12862-025-02458-9 (PMC12639931; doi:10.1186/s12862-025-02458-9)
Supplement: Supplementary file 2 — Supplementary Material 2. Model convergence. [file 12862_2025_2458_MOESM2_ESM.pdf]

Mäenpää et al. 2025. Supplementary file 2: Model convergence

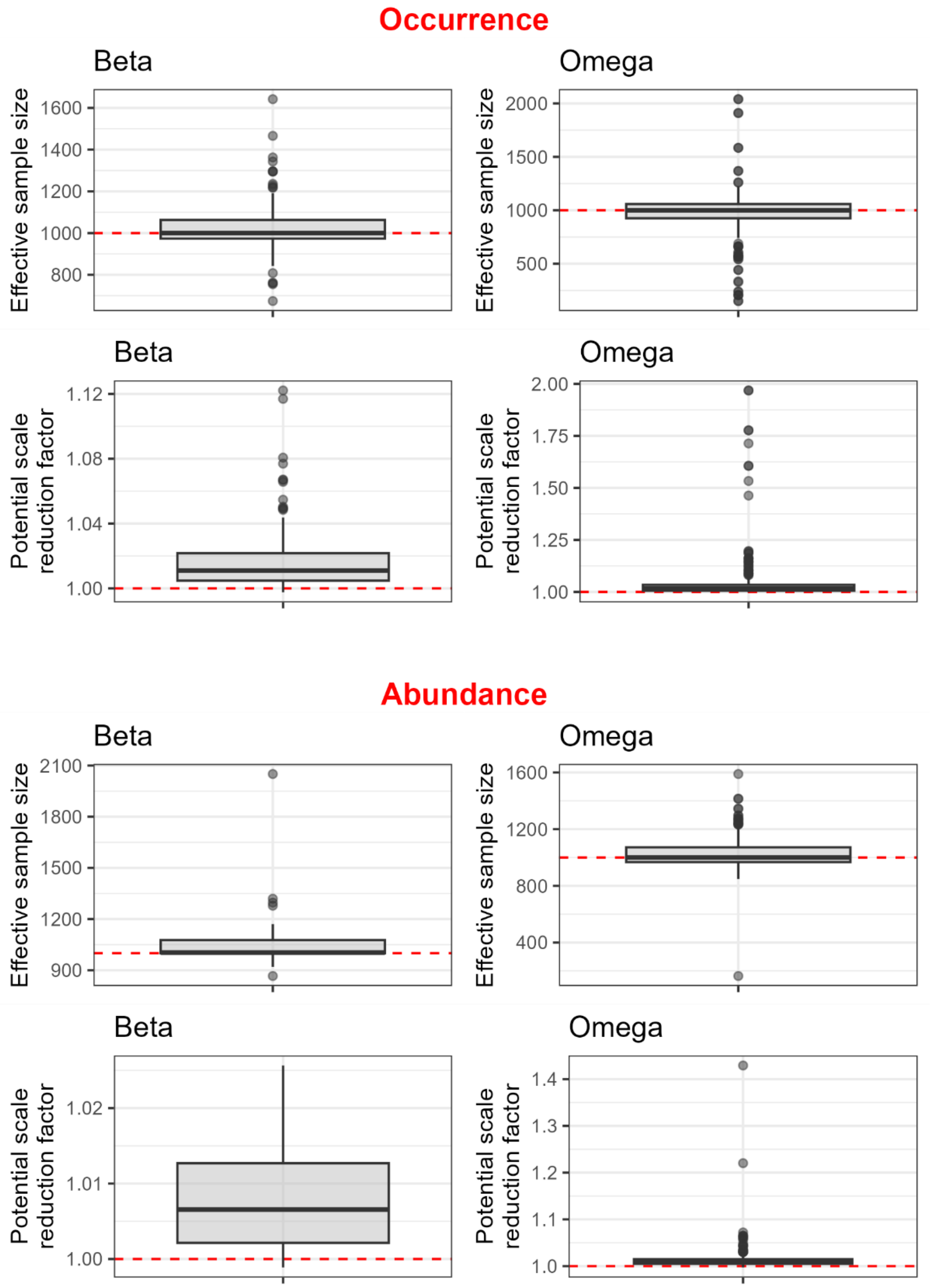

Figure S2. Estimation of the MCMC convergence for the beta (on the left side panels) and the omega (on the right side panels) parameters measured by the effective sample size and the potential scale reduction value of the occupancy model (above) and the abundance model (below). The beta parameters reflect responses to model covariates (moss weight, treatment, distance), while the omega parameters represent the residual biotic associations after accounting for fixed and random effects.
