# Supplementary material for: Tardigrade communities in pristine, drained and restored pine mire forests
Source: BMC Ecol Evol. 2025 Nov 21;25:126. doi: 10.1186/s12862-025-02458-9 (PMC12639931; doi:10.1186/s12862-025-02458-9)

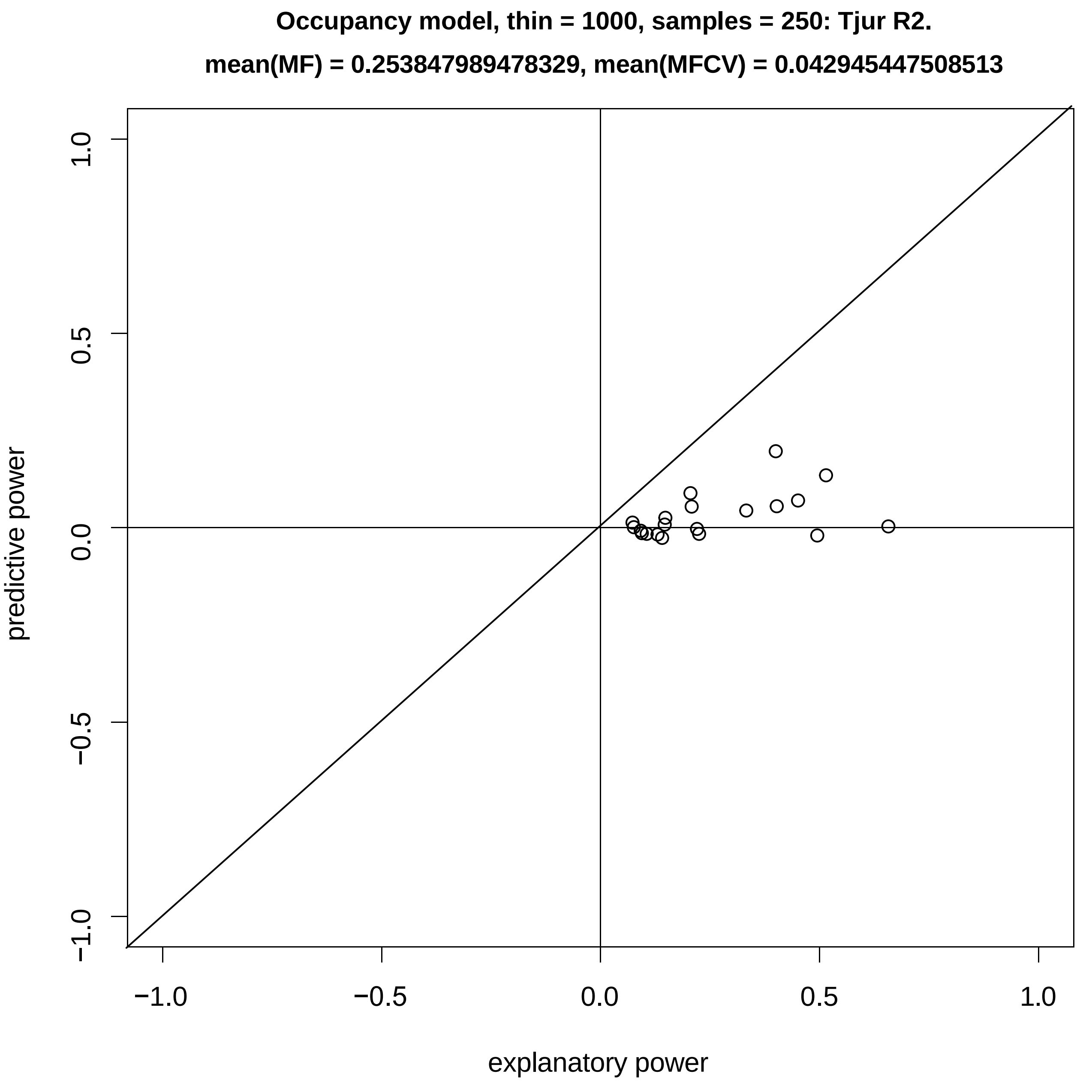
Occupancy model, thin = 1000, samples = 250: Tjur R2.
mean(MF) = 0.253847989478329, mean(MFCV) = 0.042945447508513
predictive power
explanatory power
−1.0
−0.5
0.0
0.5
1.0
−1.0
−0.5
0.0
0.5
1.0

Occupancy model, thin = 1000, samples = 250: SR2.
mean(MF) = 0.0949471664079618, mean(MFCV) = −0.0112083108274535

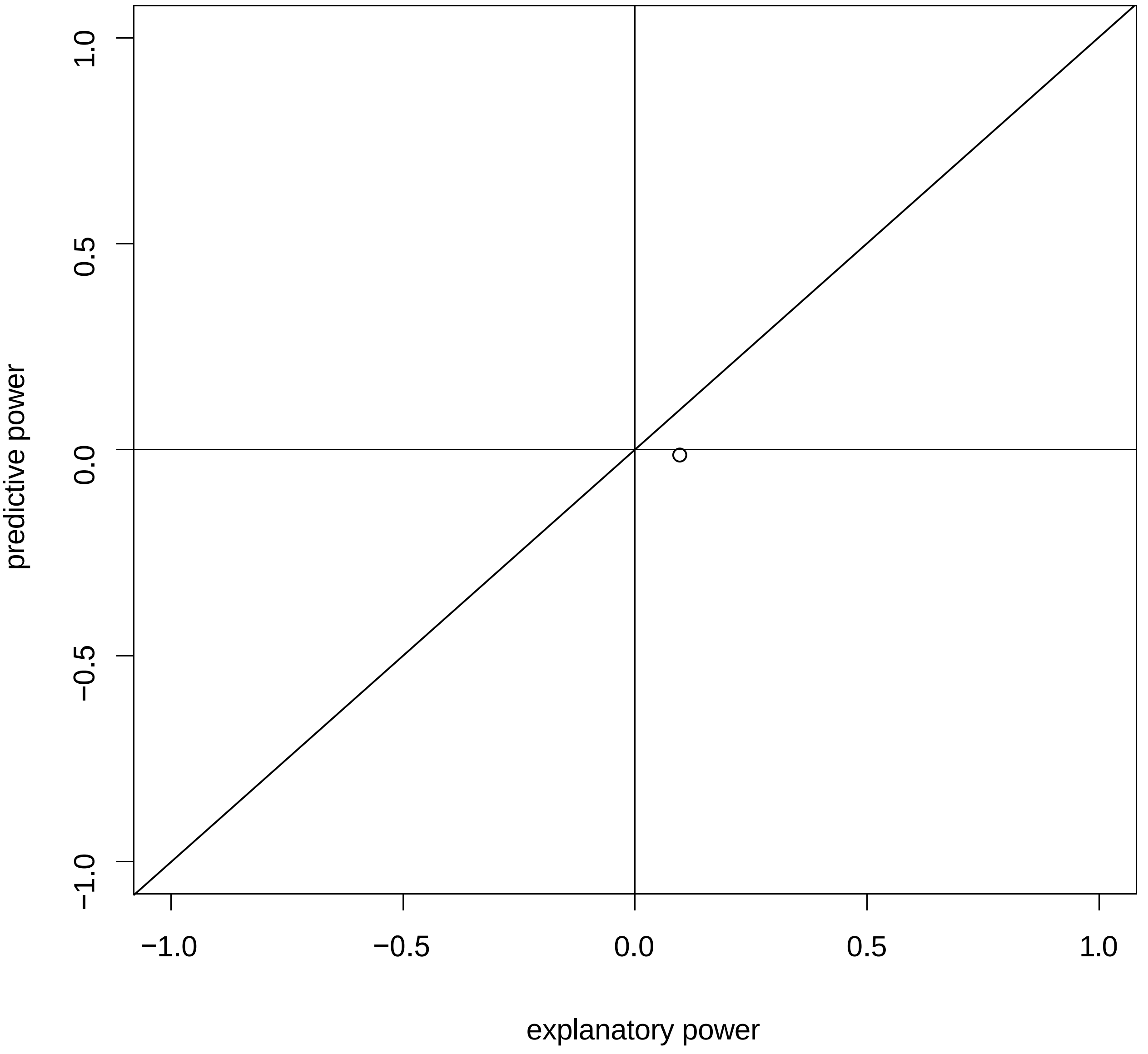

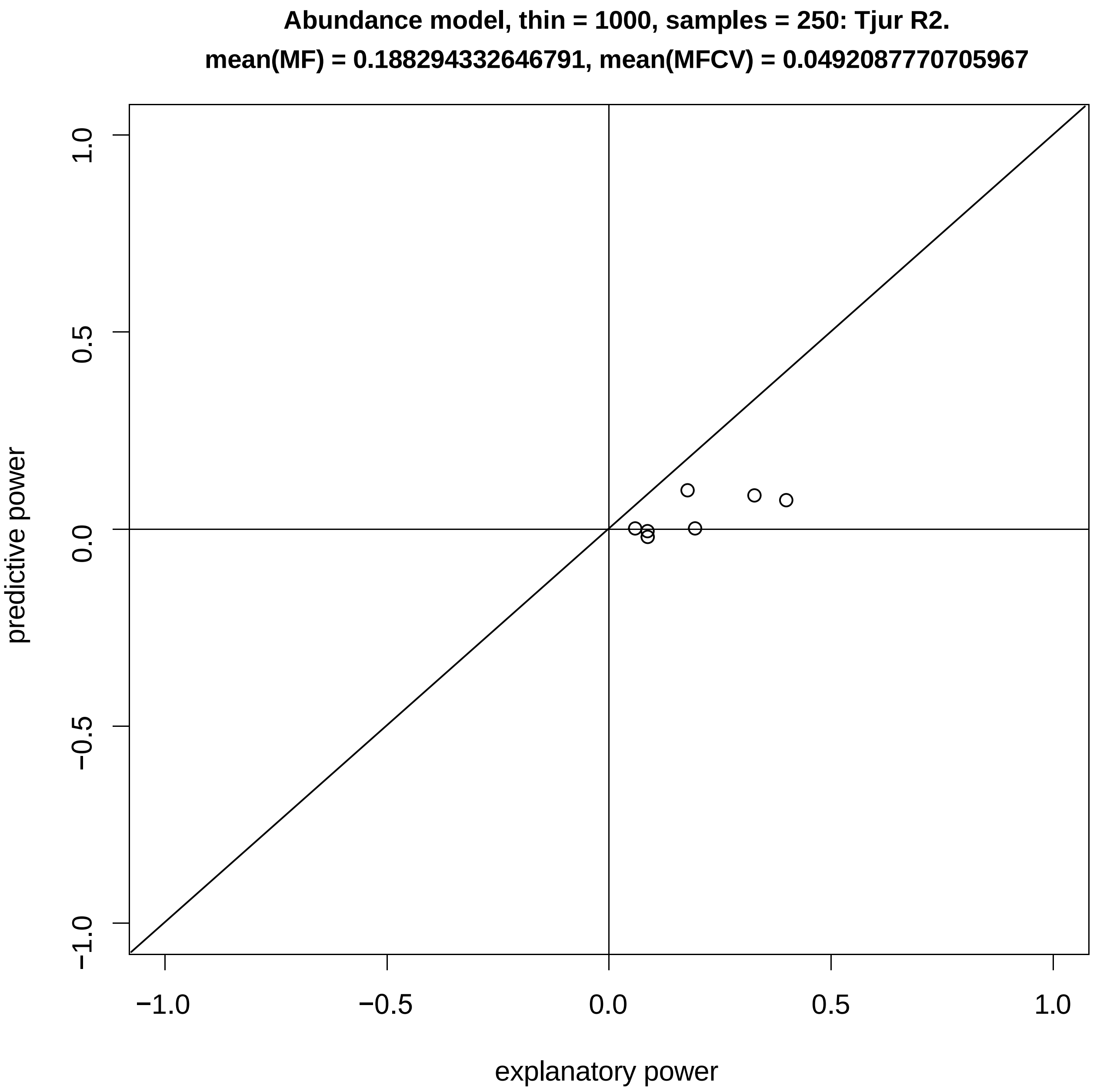
Abundance model, thin = 1000, samples = 250: Tjur R2.
mean(MF) = 0.188294332646791, mean(MFCV) = 0.0492087770705967
predictive power
explanatory power
−1.0
−0.5
0.0
0.5
1.0

Abundance model, thin = 1000, samples = 250: R2.
mean(MF) = 0.617977535800679, mean(MFCV) = −0.0613117499357741

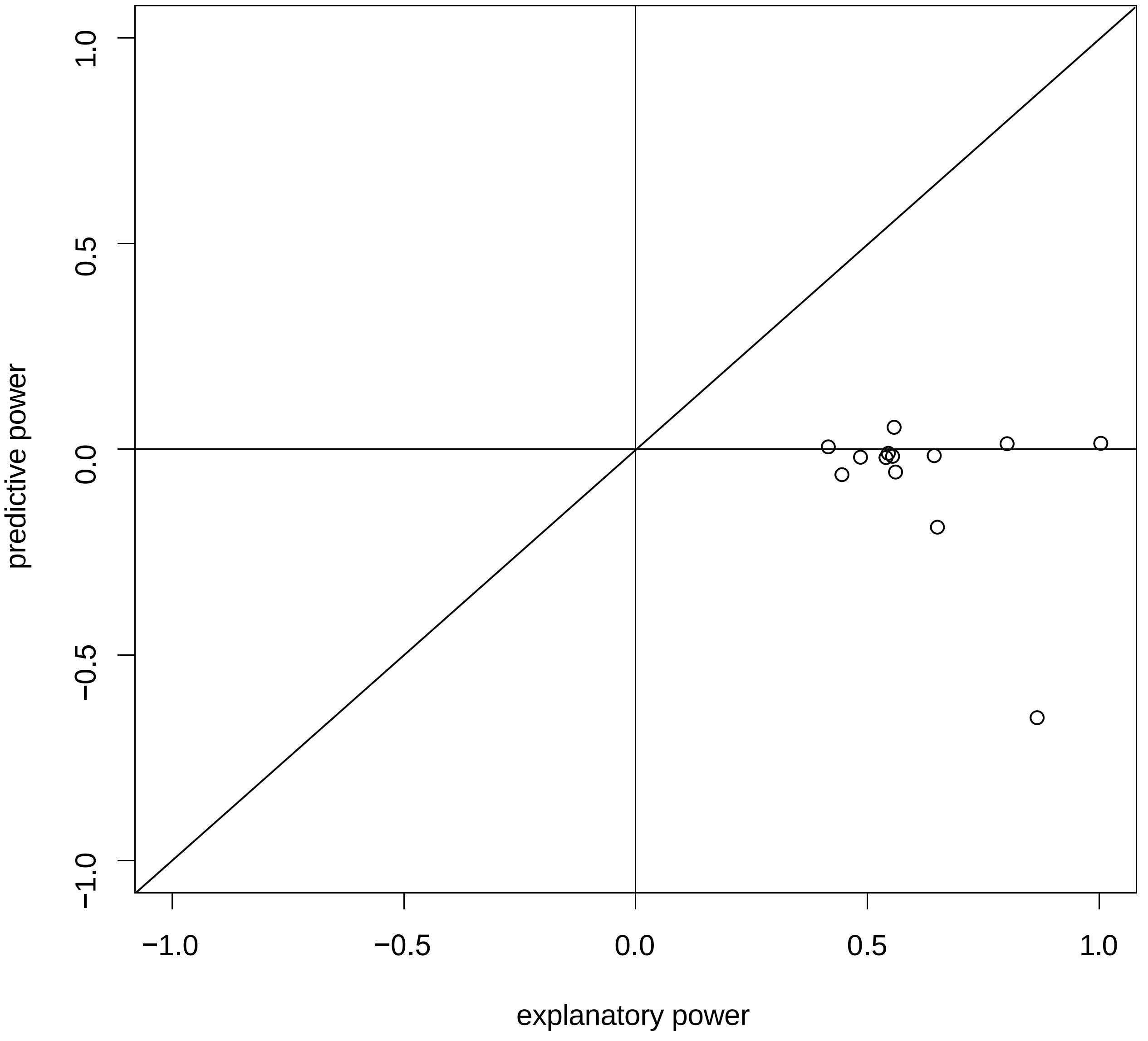

Abundance model, thin = 1000, samples = 250: SR2.
mean(MF) = 0.0866974249615752, mean(MFCV) = 1.279989286223e−05

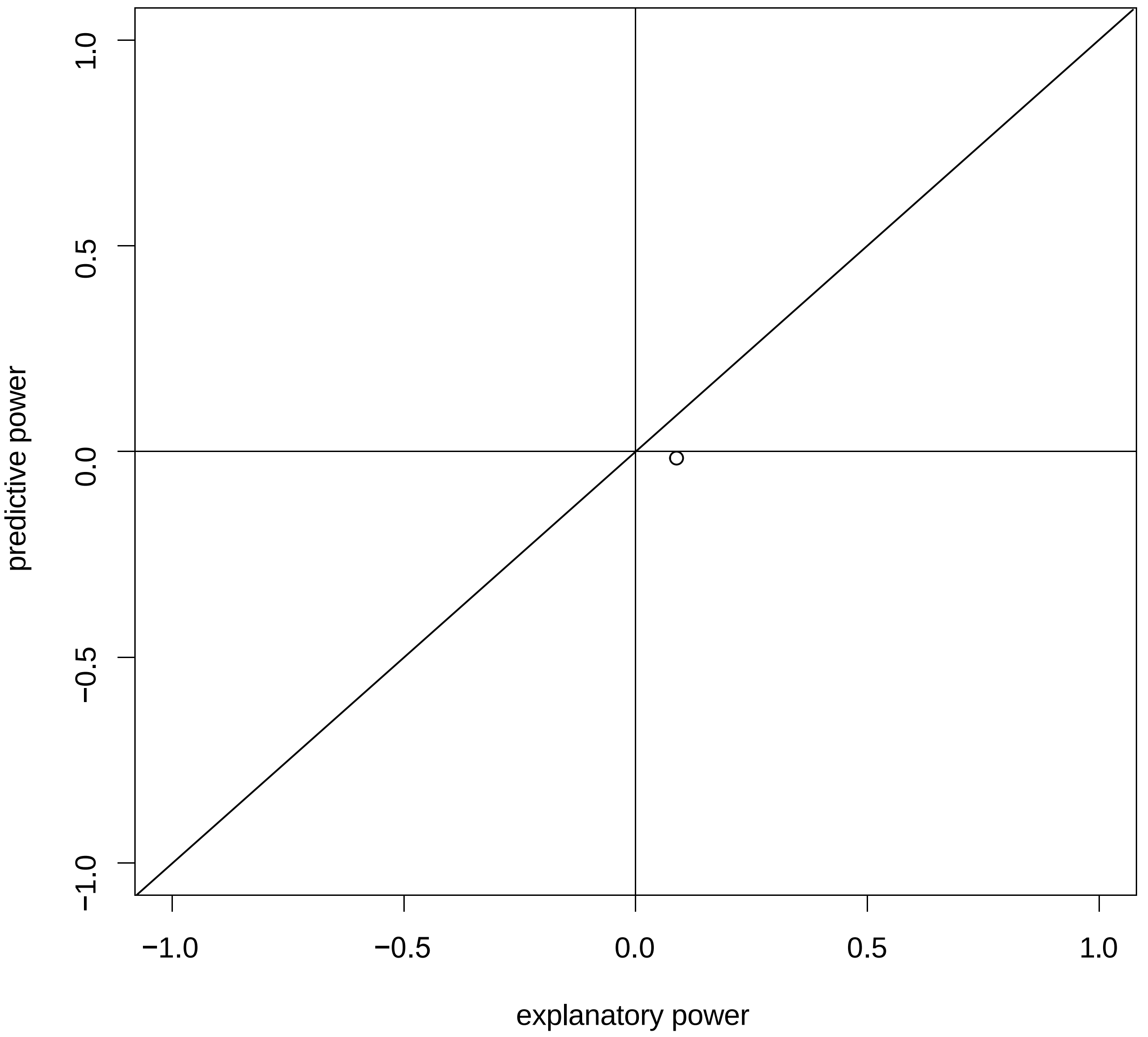

Supplement: Supplementary file 3 — Supplementary Material 3. Model two-fold cross validation. [file 12862_2025_2458_MOESM3_ESM.pdf]
