# Supplementary material for: Tardigrade communities in pristine, drained and restored pine mire forests
Source: BMC Ecol Evol. 2025 Nov 21;25:126. doi: 10.1186/s12862-025-02458-9 (PMC12639931; doi:10.1186/s12862-025-02458-9)
Supplement: Supplementary file 5 — Supplementary Material 5. Variance partition. [file 12862_2025_2458_MOESM5_ESM.pdf]

Mäenpää et al. 2025. Supplementary file 5: Variance partition of the models

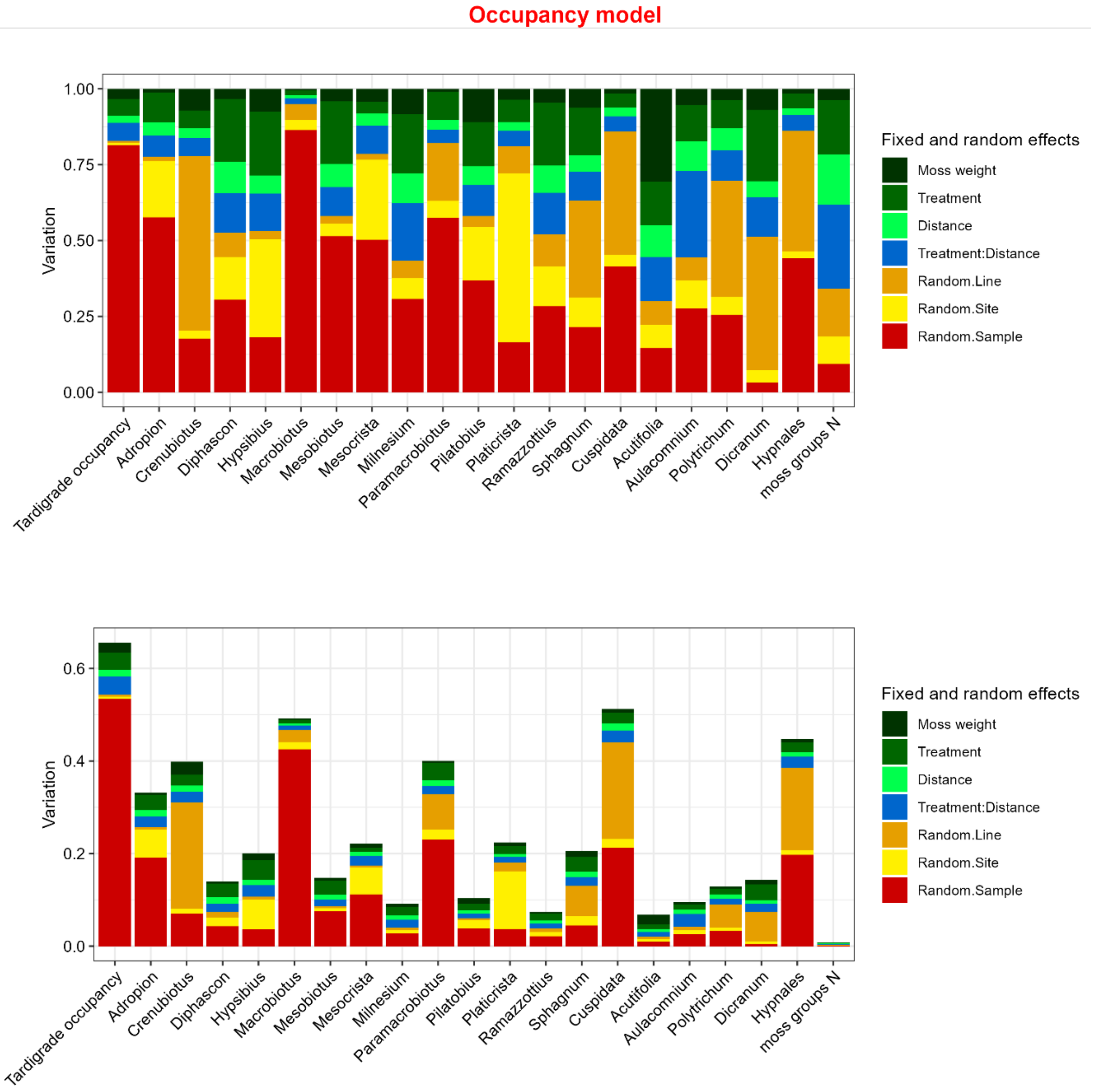

Figure S5. a) The proportion of variance explained (above) and the raw variance explained (below) by each random and fixed effect included in the occupancy model. For presence–absence responses (occupancy of tardigrades and moss types), the explained variance is quantified using Tjur's R^2 (Tjur 2009), while for count responses (number of moss groups) the explained variance is quantified using a model-based pseudo-R^2 (Ovaskainen et al. 2017). The sample ID explained 82 % of the variance in the overall tardigrade occupancy. The mean proportion of variance explained by the sample ID for all identified genera for occupancy was 40 %. However, there was a notable range across the genera in the explained proportion of occupancy (6–85 %). The spatially explicit random effect 'site ID' explained on average 11 % of the variance in occupancy which ranged from 2–19 % across individual genera. Among the fixed effects treatment and interaction of treatment and distance from the center of the peatland explained more of the variance than moss weight. 5 % of the overall tardigrade occupancy

was explained by treatment. Across individual tardigrade genera treatment explained on average 13 % (1–21 %), whereas the interaction of treatment and distance explained on average 9 % (2–19 %) of the variation in occupancy.

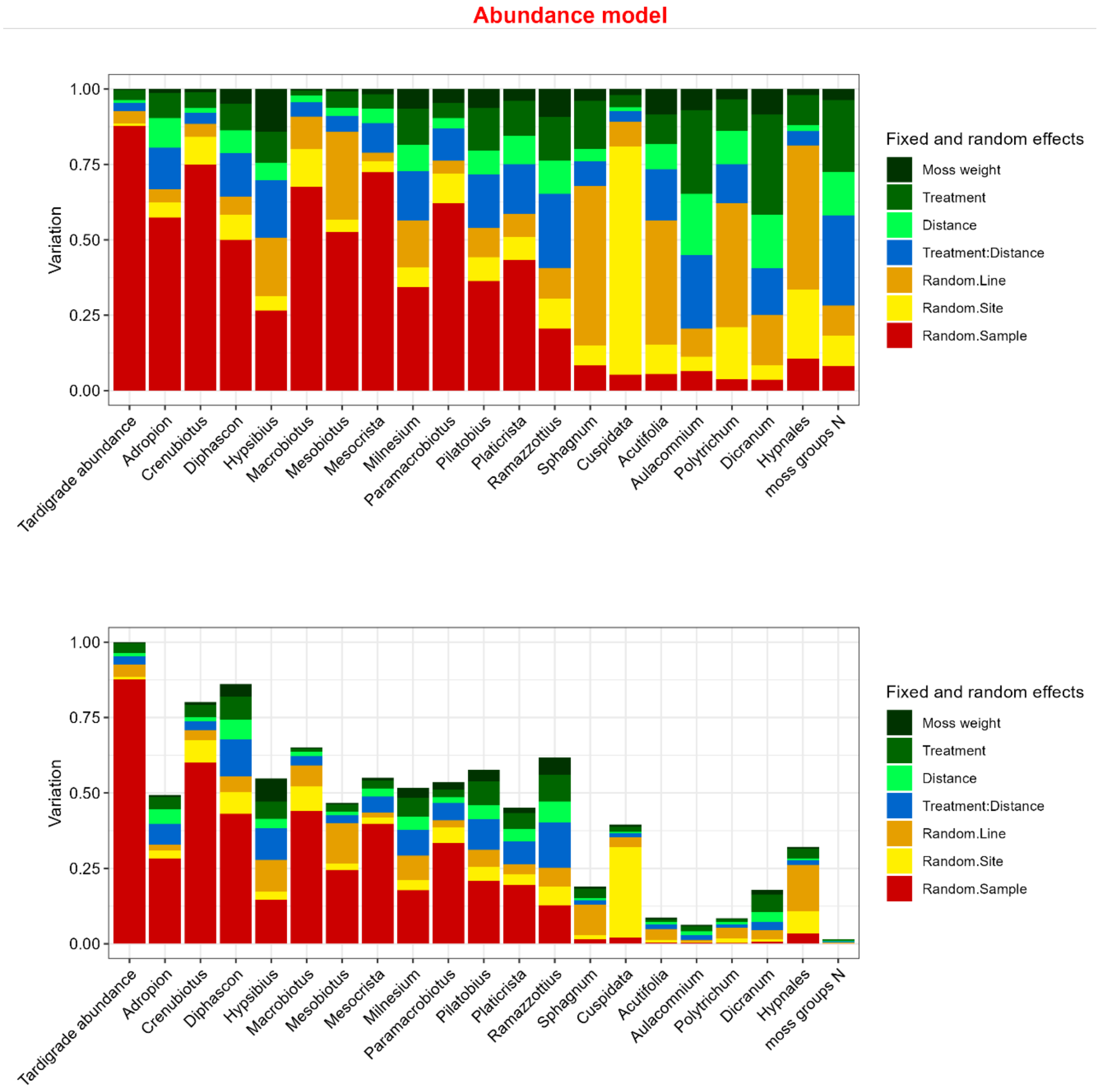

Figure S5. b) The proportion of the variance explained (above) and the raw variance explained (below) by each random and fixed effect included in the abundance model. For continuous responses (tardigrade abundance) the explained variance is quantified using R^2, for presence–absence responses (moss types), the explained variance is quantified using Tjur's R^2 (Tjur 2009), and for count responses (number of moss groups) it is quantified using a model-based pseudo-R^2 (Ovaskainen et al. 2019). The sample ID explained 87 % of the variation in tardigrade overall abundance. The mean proportion of

variance explained by the sample ID for all identified genera for abundance was 49 % and the range across the genera was 20–75 %. The spatially explicit random effect 'site ID' explained on average 10 % of the variance in abundance of individual genera ranging from 4–12 %. 3 % of the overall abundance was explained by treatment. Across individual tardigrade genera treatment explained on average 9 % (2–15 %), whereas the interaction of treatment and distance explained on average 13 % (4–25 %) of the variance.

References

Tjur, T. (2009). Coefficients of determination in logistic regression models – a new proposal: The coefficient of discrimination. *The American Statistician*, 63(4), 366–372.

Ovaskainen, O., Tikhonov, G., Norberg, A., Guillaume Blanchet, F., Duan, L., Dunson, D., Roslin, T., & Abrego, N. (2017). How to make more out of community data? A conceptual framework and its implementation as models and software. *Ecology Letters*, 20(5), 561–576.
